# Core interventions contributing to the effectiveness of the National Urban Health Mission in India

**DOI:** 10.7189/jogh.13.03009

**Published:** 2023-03-10

**Authors:** Snehashish Raichowdhury, Sonalini Khetrapal, Brian Chin

**Affiliations:** 1 Cerulean Consulting, Kolkata, India; 2 Asian Development Bank, Manila, Philippines

**Figure:**
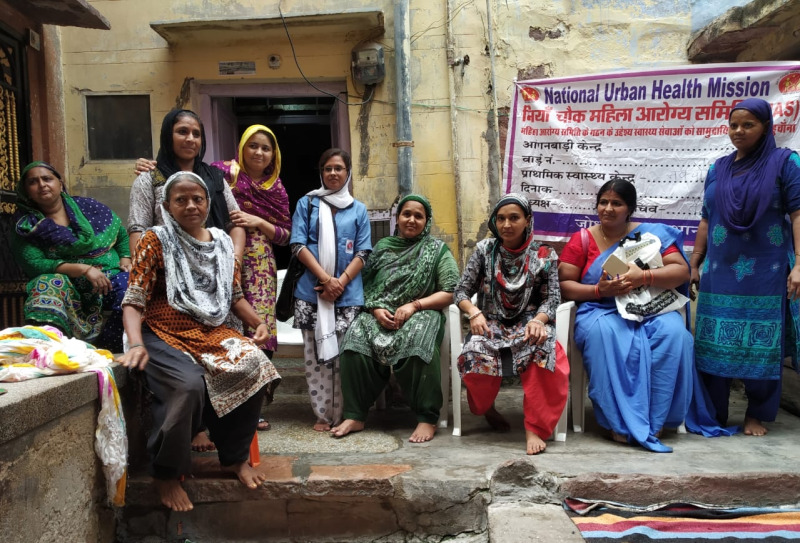
Photo: Mahila Aarogya Samitis (MAS) meeting between the members and Urban Accredited Social Health Activist (ASHA) in Jodhpur, India. Source: Sonalini Khetrapal, used with permission.

The National Urban Health Mission (NUHM) was launched by the Government of India as a sub-mission of the National Health Mission (NHM) in May 2013, and was the first major programmatic response at the national level to address the health issues of the urban poor [1]. With around 38.6% of the population (estimated at 479 million in 2022) expected to live in urban areas by 2036 [2], providing health care services to this city-based population will largely determine India’s success in achieving universal health care [3]. The existing primary health facilities, grossly inadequate in number, varied in norms and quality, and had limited scope of services. Absence of a comprehensive health system in urban areas prior to NUHM resulted in confusion and increased inequalities, especially among the poor and migrant population [4].

We analysed data from programme implementation, as well as government reports and data available in the public domain, from the period of 2014-2022 to highlight some of NUHM’s key achievements, while identifying a few areas that need strengthening [5,6].

## CORE INTERVENTIONS IN NUHM

NUHM was conceived as a truly holistic, comprehensive, and multidimensional programme with the urban poor in focus. It included several layers of core interventions, termed as “key thrust areas” by the Ministry of Health and Family Welfare (MOHFW), that could be clustered under eight topics [7].

### Planning and management

NUHM consisted of one Urban Community Health Centre (UCHC) for a population of 500 000 in metro cities and 250 000 for smaller cities; and one Urban Primary Health Centre (UPHC) for every 50 000 residents. Frontline workers like an auxiliary nurse midwife (ANM) for every 10 000 residents and a female volunteer or accredited social health activist (ASHA) are covering a population of up to 2500 conducted outreach services. The municipal corporations of seven megacities of India (Mumbai, Delhi, Kolkata, Chennai, Hyderabad, Bangalore, and Ahmedabad) were responsible for implementing NUHM. For the smaller towns, the state health department had oversight of implementation of NUHM. This is a unique arrangement launched in 779 cities [1] and recognizes the differential institutional strengths of these urban local bodies (ULBs) appropriately.

NUHM functionaries were trained on a wide range of topics including technical subjects, management development, communication and behaviour change, and intersectoral convergence. Despite challenges pertaining to retention of staff, a total of 8609 medical officers, 7634 staff nurses, 8031 ANMs, 1712 programme management staff, and 41 442 ULB staff were trained in various aspects until September 2022 [8].

### Urban Primary Health Centres

NUHM focused on serving the urban poor through 5025 functional UPHCs between 2014 and 2022 [8]. Compared to a total of 871 Urban Health Posts per 50 000 urban population existing mostly in India’s metros before NUHM [9], the much better equipped and staffed UPHCs funded by NUHM are now distributed more equitably all over the nation. Facility and slum mapping were completed in 1100 and 1094 of the 1178 cities respectively, while 826 cities completed vulnerable population groups mapping until September 2022 [8].

### Outreach services

Community outreach through Urban ASHAs and community women collectives called Mahila Aarogya Samitis (MAS) have helped generate demand for preventive services and increased access to facility level care. By September 2022, 78 127 ASHAs were deployed by MOHFW and 80 852 MAS were formed to support their respective catchment populations [8].

### Quality assurance

Under NHM, the State Quality Assurance Committee and State Quality Assurance Units have been set up in all states and union territories. The National Quality Assurance Standards (NQAS) are recognized internationally and have received accreditation from the International Society for Quality in Health Care (ISQua). As of June 2022, 150 UPHCs have been NQAS certified [10]. The Government of India also initiated a patient satisfaction system named “Mera Aspataal” (My Hospital) to empower patients to express their views on health services.

### Monitoring and evaluation

The implementation of NUHM has been strengthened by establishing programme management units (PMUs) at state, district and city levels. Initiatives were taken to disaggregate urban health data in the Health Management Information System (HMIS) and coordinate with key schemes under the Reproductive and Child Health programme. This resulted in the improvement in quality of services, better coordination between urban health functionaries and community level workers, and increased outreach activities.

### Public-private partnerships and innovations

NUHM has developed a framework for innovations and partnerships in 2018 [11] and documented models implemented in 20 states. It has explored public-private partnerships for: making ward level convergence committees and partnerships with non-governmental organisations in Odisha; electronic or e-UPHCs in Andhra Pradesh to bring essential as well as specialist services (e.g. cardiology) to the slum dwellers by harnessing technology; and Basti Dawakhana (slum clinic) in Telangana to provide free outpatient services to the urban poor. It is crucial for such models to be adopted and assume a bigger scale to further influence health actions.

### Intersectoral convergence for urban local bodies

NUHM led to initiatives for increased coordination in the states for intersectoral convergence; strengthened integration with vertical programmes such as the Integrated Disease Surveillance Programme (IDSP); and increased coordination with programmes like the National Urban Livelihood Mission (NULM) and Swachh Bharat Mission (Clean India Mission). In addition, a national convergence framework was developed in 2019 [12] and support for the implementation of few convergent actions (vector control, community processes, epidemic management, and water and sanitation) were provided.

### Finance

The actual expenditure on NHM increased by 64% between 2016-17 and 2020-21, while that of the MOHFW more than doubled during the same period [13]. However, financing of NUHM remained stagnant over the years, budgeted at Indian rupees (INR) 9500 million in financial years 2019-20 and 2020-21, increasing only from INR 8680 million in 2018-19 [14]. Though NHM is the largest component of the expenditure incurred by MOHFW, its share in total expenditure of the ministry fell from 58.6% in 2016-17 to 46.4% in 2020-21. An inadequate allocation for health in states will keep posing challenges to improve primary health care [3,15].

## CONTRIBUTIONS OF NUHM TO HEALTH SYSTEMS AND OUTCOMES

Implementation of NUHM has provided the urban poor with an increase in access to health care as well as a choice to select public sector facilities over the costly private sector. Data from 2014 and 2017-18 (**Table 1**) show ailments treated by the private sector in urban areas reduced by 11% (i) while access to outpatient department (OPD) services in urban government facilities increased by 19% (ii) [16,17]. The hospitalization rate per 1000 people in all urban facilities in a year reduced by 23% (iii), while the percentage of people hospitalized in urban public facilities increased by 10% (iv), suggesting higher benefits accruing from the NUHM-funded facilities. The reduction in the hospitalization rate may be partly attributed to better primary health care in both public and private facilities, resulting in lesser need for hospitalization.

**Table 1:** Urban health services in 2014 and 2017-18 from National Sample Surveys

Description		2014 (NSS – 71^st^ round)	2017-18 (NSS – 75^th^ round)	Difference (%)
**(i)**	Percentage of ailments treated at private doctors/clinics (non-hospital)	50.0	44.3	-11%
**(ii)**	Percentage of ailments treated at government hospitals (OPD) and other public facilities	21.2	26.2	19%
**(iii)**	Number hospitalized (excluding childbirth) per 1000	44.0	34.0	-23%
**(iv)**	Percentage of hospitalization in government hospitals (excluding childbirth)	32.0	35.3	10%
**(v)**	Average medical expenditure (in INR) for non-hospitalization cases in allopathic treatment in public hospitals	639.0	344.0	-46%
**(vi)**	Percentage of the lowest quintile class of household expenditure covered by health insurance	8.6	9.8	14%

NSS – National Sample Survey, OPD – outpatient department, INR – Indian rupees

Reducing out-of-pocket expenditure (OOPE) by almost 46% for non-hospitalization cases in urban government hospitals (v), especially with urban poor having health insurance coverage below 10% (vi), is a major contribution of NUHM. It is also important to note that the average total medical expenditure, contributed largely by medicines, is about one-third of what is charged in private hospitals for the same services (INR 344 vs. INR 1038) [17].

NUHM’s contribution to reproductive health outcomes include increase in institutional birth in urban government facilities from 42.0% to 48.3% for all and 54.0% to 62.1% for the poorest quintile between 2014 and 2017 [16,17]. The percentage of women receiving antenatal and postnatal care also increased to 98.0% from 92.9% and 90.0% from 84.1% respectively during the same period [16,17].

## CONCLUSION

Apart from increasing institutional deliveries in urban areas, NUHM has contributed to increased utilization of services, reduced OOPE for health care of the urban poor, and reduced disparities in urban health care infrastructure in bigger and smaller towns. Given its initial success, NUHM needs to build on the momentum with more financing and strengthen mechanisms for partnerships, governance, and intersectoral convergence to further improve health outcomes of the urban poor.
